# Parasite clearance, cure rate, post-treatment prophylaxis and safety of standard 3-day versus an extended 6-day treatment of artemether–lumefantrine and a single low-dose primaquine for uncomplicated *Plasmodium falciparum* malaria in Bagamoyo district, Tanzania: a randomized controlled trial

**DOI:** 10.1186/s12936-020-03287-5

**Published:** 2020-06-23

**Authors:** Lwidiko E. Mhamilawa, Billy Ngasala, Ulrika Morris, Eliford Ngaimisi Kitabi, Rory Barnes, Aung Paing Soe, Bruno P. Mmbando, Anders Björkman, Andreas Mårtensson

**Affiliations:** 1 Department of Women’s and Children’s Health, International Maternal and Child Health (IMCH), Uppsala University, Uppsala, Sweden; 2 Department of Parasitology and Medical Entomology, Muhimbili University of Health and Allied Sciences, Dar es Salaam, Tanzania; 3 Department of Microbiology, Tumor and Cell Biology, Karolinska Institutet, Stockholm, Sweden; 4 Office of Clinical Pharmacology, Division of Pharmacometrics, Food and Drugs Administration, Silver Spring, MD USA; 5 Tanga Centre, National Institute for Medical Research, Tanga, Tanzania

**Keywords:** Malaria, *Plasmodium falciparum*, Artemether–lumefantrine, Drug resistance, Tanzania

## Abstract

**Background:**

Artemisinin-based combination therapy (ACT) resistant *Plasmodium falciparum* represents an increasing threat to Africa. Extended ACT regimens from standard 3 to 6 days may represent a means to prevent its development and potential spread in Africa.

**Methods:**

Standard 3-day treatment with artemether–lumefantrine (control) was compared to extended 6-day treatment and single low-dose primaquine (intervention); in a randomized controlled, parallel group, superiority clinical trial of patients aged 1–65 years with microscopy confirmed uncomplicated *P. falciparum* malaria, enrolled in Bagamoyo district, Tanzania. The study evaluated parasite clearance, including proportion of PCR detectable *P. falciparum* on days 5 and 7 (primary endpoint), cure rate, post-treatment prophylaxis, safety and tolerability. Clinical, and laboratory assessments, including ECG were conducted during 42 days of follow-up. Blood samples were collected for parasite detection (by microscopy and PCR), molecular genotyping and pharmacokinetic analyses. Kaplan–Meier survival analyses were done for both parasite clearance and recurrence.

**Results:**

A total of 280 patients were enrolled, 141 and 139 in the control and intervention arm, respectively, of whom 121 completed 42 days follow-up in each arm. There was no difference in proportion of PCR positivity across the arms at day 5 (80/130 (61.5%) vs 89/134 (66.4%), p = 0.44), or day 7 (71/129 (55.0%) vs 70/134 (52.2%), p = 0.71). Day 42 microscopy determined cure rates (PCR adjusted) were 97.4% (100/103) and 98.3% (110/112), p = 0.65, in the control and intervention arm, respectively. Microscopy determined crude recurrent parasitaemia during follow-up was 21/121 (17.4%) in the control and 14/121 (11.6%) in the intervention arm, p = 0.20, and it took 34 days and 42 days in the respective arms for 90% of the patients to remain without recurrent parasitaemia. Lumefantrine exposure was significantly higher in intervention arm from D3 to D42, but cardiac, biochemical and haematological safety was high and similar in both arms.

**Conclusion:**

Extended 6-day artemether–lumefantrine treatment and a single low-dose of primaquine was not superior to standard 3-day treatment for ACT sensitive *P. falciparum* infections but, importantly, equally efficacious and safe. Thus, extended artemether–lumefantrine treatment may be considered as a future treatment regimen for ACT resistant *P. falciparum*, to prolong the therapeutic lifespan of ACT in Africa.

*Trial registration* ClinicalTrials.gov, NCT03241901. Registered July 27, 2017 https://clinicaltrials.gov/show/NCT03241901

## Background

Historically, *Plasmodium falciparum* resistance to anti-malarial drugs, e.g., chloroquine and sulfadoxine–pyrimethamine, has evolved in South-East Asia, and spread via the Indian sub-continent to Africa [1] with devastating effects on malaria case management on the African continent [2, 3]. During the past 10–15 years artemisinin-based combination therapy (ACT) has replaced mono-therapies as first-line treatment for uncomplicated *P. falciparum* malaria in Africa, and significantly contributed to the decline in malaria burden [4]. However, progress made in malaria control has currently stalled, and in the 2017–2018 period, there was an increase of 3.5 million new malaria cases in 10 highest burden countries in Africa including Tanzania [4, 5]. Moreover, the development of artemisinin resistance in South East Asia has spread westward since 2009, reaching eastern India in 2019 [6–8]. This makes it urgent to develop and scientifically evaluate strategies with the potential to protect the therapeutic lifespan of ACT as lifesaving drugs in Africa.

Artemisinin resistance, defined as partial resistance by the WHO, is phenotypically characterized by prolonged *P. falciparum* clearance time after ACT (microscopy positivity rate on day 3 of ≥ 10%) [9]. Resistance has been linked to specific mutations in the *P. falciparum kelch 13* propeller gene (*pfk13*) [7, 9–12]. Such mutations have recently been documented in Rwanda, in the first report of locally arising *pfk13* mutations in Africa, although without affecting the efficacy of artemether–lumefantrine, the most commonly used ACT in Africa [5]. From the mutations reported in Rwanda, one was among validated markers of artemisinin resistance (561H) and three were candidate markers (469F, 441L and 449A) [13].

Although microscopy determined parasite clearance and overall average therapeutic efficacy rates has remained above 98% across Africa for artemether–lumefantrine [4, 14, 15], concerns have lately arisen on the future long-term efficacy of artemether–lumefantrine in Tanzania, where this ACT has been used as first line treatment for uncomplicated *P. falciparum* malaria since 2006. Several observations from Bagamoyo district contribute to this concern. Firstly, there are reports of polymerase chain reaction (PCR) determined positivity rate on day 3 in the magnitude of 28–84% after supervised artemether–lumefantrine treatment [16, 17]. Using a deep sequencing approach, some of these *P. falciparum* sub-populations showed microscopy determined parasite clearance curves similar to artemisinin resistant parasites in Cambodia [18]. Secondly, molecular epidemiological studies from Bagamoyo district, have shown temporal selection of single nucleotide polymorphisms (SNP) linked to lumefantrine tolerance/resistance in the *P. falciparum* population, without compromised cure rates [19]. These SNPs include the N86 (most strongly linked to lumefantrine tolerance), 184F and D1246 in the *P. falciparum* multidrug resistance 1 (*pfmdr1*) gene and, the K76 SNP in the *P. falciparum* chloroquine resistance transporter (*pfcrt)* gene [19–22]. During reinfection, parasites with selected tolerance/resistance associated genotypes (NFD), have shown to be able to withstand 15-fold higher lumefantrine blood concentrations than those with the alternative haplotype (YYY) [23]. The selection of such parasites may in turn lead to a gradually shortened post-treatment prophylactic period, long before clinical treatment failures are apparent. Together, these observations may represent early warning signs of ACT resistance in Tanzania.

A potential strategy to protect the therapeutic life span of ACT, in an era of imminent risk of ACT resistance, would be to extend treatment duration. Dosage prolongation of artemether–lumefantrine from 2 to 3 days has been previously implemented to prevent treatment failures and resistance development from suboptimal dosing [24, 25]. Presently there is evidence of under-dosing among pregnant women from second trimester and above, and malnourished African children [26, 27]. These vulnerable groups are at higher risk of serious malarial disease and importantly, may be more likely to foster resistance development due to sub-therapeutic exposure of parasites to the drug. Further dose prolongation of up to 5 days has been suggested for these groups based on in silico dose optimization [28]. However, studies from Africa are needed to evaluate efficacy, safety and tolerability of dose prolongation for the treatment of uncomplicated malaria. To date, there are two published studies on artemether–lumefantrine dosage extension to 5 days; one from Myanmar on non-pregnant adults and children [29], and the other on pregnant vs non-pregnant women in Congo [30], as the only study from Africa to evaluate artemether–lumefantrine dosage extension.

In this study, the efficacy and safety of doubling the dose, i.e., from 3 to 6 days, of artemether–lumefantrine was evaluated, since this is logistically pragmatic and feasible to implement with the current packaging of the drug. Additionally, a 6-day artemether–lumefantrine treatment regime will cover three erythrocytic cycles with adequate concentration of the artemisinin component to kill parasites demonstrating a delayed clearance. Moreover, the World Health Organization (WHO) recommends use of single low-dose primaquine (0.25 mg/kg), a *P. falciparum* gametocytocidal drug; for blocking transmission in low-transmission areas in combination with ACT irrespective of glucose-6-phosphate dehydrogenase enzyme status [4]. Gametocytes arising from asexual parasites able to withstand prolonged artemether–lumefantrine treatment might represent resistant prone sub-populations. Such parasite sub-populations might be of key importance to eliminate in order to delay *P. falciparum* drug resistance development and spread [31–33]. Previous data on the safety of single low-dose primaquine in Bagamoyo district is available [34]. However, its addition to the first dose of artemether–lumefantrine did not influence gametocyte clearance [35]. In this study, single dose of 0.25 mg/kg primaquine was, therefore, added on the last day of extended artemether–lumefantrine treatment, which is also supported by modelling data [36].

## Methods

### Aim

The overall aim of this study was to compare PCR-determined parasite clearance, cure rate, post-treatment prophylaxis and safety, of standard treatment (3 days) versus an extended treatment (6 days) of artemether–lumefantrine with single low-dose primaquine for uncomplicated *P. falciparum* malaria in Bagamoyo district, Tanzania.

### Study design

This was a two-armed, randomized controlled, parallel group, superiority clinical trial, with allocation ratio 1:1. Blinding to treatment arm allocation was done to investigators and staff who were not involved in study drug administration. The first day of screening and enrollment is referred to as day 0 (D0), with D0I referring to screening sample time point, and D0II as enrolment sample time point and D0III as second visit of day 0. For subsequent visits from day 1 (D1) onwards, AM was used to refer to the first visit of the day and PM referring to the second visit. The study was conducted between July 2017 and March 2018. The study has been registered at clinicaltrials.gov (identifier: NCT03241901).

### Study outcomes

The primary outcome was the proportion of PCR detectable *P. falciparum* on D5 and D7 in the respective arms. Secondary outcomes included fever and microscopy parasite clearance times, crude and PCR corrected cure rates, post treatment prophylaxis, selection of genetic drug resistance markers and safety.

### Sample size calculation

The sample size was calculated based on an assumed clinically meaningful difference between the arms in PCR determined *P. falciparum* positivity rate of 15% on D5 (20% and 5%, in the control and intervention arm respectively). There was no data available on PCR positivity rates beyond day 3 from the study site. PCR positivity rates were therefore predicted, based on the day 3 PCR positivity rates from previous studies [16], to decline on days 5 and 7. It was assumed that the extended treatment arm would have a lower prevalence compared to control arm due to more effective parasite clearance. To be able to show this difference with 90% power at 0.05 significance level and allowing for 20% attrition, 140 patients were required in each arm.

### Randomization

Prior to commencement of the study, computer-based randomization was done using R studio program (RStudio, Inc, Boston, Massachusetts), version 1.1.456. A randomization list stratified by study site was generated by a statistician for both treatment arms. For allocation concealment, opaque envelopes were serially numbered and treatment arm allocation cards were inserted according to the list. The envelopes were sealed and the study nurse who was responsible for patient enrollment wrote the unique patient ID on top of the sealed envelope. This envelope was opened by the study clinician only, to determine the treatment arm allocation and for dispensing study drugs.

### Study area

The study was conducted in Bagamoyo district, Coast region, Tanzania. Malaria transmission in Bagamoyo district is considered moderate, occurring throughout the year with peaks related to the long rainy season in May to July, and short rains in November to December [37]. *P. falciparum* is the predominant malaria species [38].

Yombo and Fukayosi public primary health care facilities were purposely selected as study sites. These rural sites provide basic health care services for the residents in their respective catchment area and have served as research sites for previous studies [16, 19]. The facilities have laboratory capacity to carry out malaria rapid diagnostic tests and microscopy. During the study period, there was an ongoing campaign for malaria control through mosquito nets distribution around Yombo facility. In 2017, Bagamoyo district had the highest insecticide treated nets (ITN) ownership (89%) across all regions of mainland Tanzania [39].

### Study participants

#### Screening

All patients presenting at the health care facilities with fever (defined as axillary temperature ≥ 37.5 °C) or history of fever in the last 24 h were screened using a malaria rapid diagnostic test (RDT) (CareStart™ Malaria Pf/PAN (HRP2/pLDH) Ag RDT, Access Bio, Inc. NJ, USA). Thick and thin blood films were obtained for microscopy determined parasite counts and species identification from RDT positive patients, who were then screened for study eligibility.

#### Inclusion and exclusion criteria

Participants providing written informed consent were eligible if they had microscopy confirmed *P. falciparum* mono-infection irrespective of parasite density, were between 1 and 65 years old, with a body weight > 10 kg, and electrocardiogram (ECG) determined QTc interval between 360 and 440 ms in males and 370–460 ms in females.

Individuals were excluded from enrollment if they had symptoms/signs of severe illness, severe malnutrition, were pregnant, breastfeeding, or unwilling to practice birth control during participation in the study, had haemoglobin levels < 8 g/dL, were allergic to the study medications or on regular medication which may interfere with anti-malarial pharmacokinetics, reported anti-malarial intake within the last 2 weeks, or had had a blood transfusion within the last 90 days.

### Study intervention

Patients allocated to the control arm received standard treatment, i.e., a weight-based, 3-day course of artemether–lumefantrine 20/120 mg/kg (Coartem^®^, Novartis Pharma, Basel, Switzerland), administered twice a day (at 0, 8, 24, 36, 48 and 60 h), as per Tanzania national treatment guidelines for uncomplicated *P. falciparum* malaria [40]. In the intervention arm, patients received a prolonged, weight based, 6-day course (at 0, 8, 24, 36, 48 60, 72, 84, 96, 108, 120, and 132 h) of artemether–lumefantrine 20/120 mg/kg, together with single low-dose primaquine phosphate 0.25 mg/kg (Neo-Quipenyl^®^, Sanofi-aventis Canada Inc.) administered with the last dose of artemether–lumefantrine. All patients received directly observed therapy for all drug doses. Children unable to swallow solid artemether–lumefantrine tablets received dispersible tablets suspended in water. Primaquine was administered by suspending 15 mg tablets in 15 mL of water and measured using a sterile syringe as previously described [34].

Patients were encouraged to eat after every drug intake, based on previous studies confirming that fat content in a standard African meal is sufficient to achieve adequate plasma concentrations of lumefantrine [41, 42]. It was explained to all participants that food intake enhances absorption of artemether–lumefantrine and reduces the gastrointestinal side effects of primaquine. After each drug dose, patients were observed for 30 min, and treatment was re-administered in case of vomiting.

### Patient follow-up

Clinical and laboratory assessments were performed after 8 h on D0, thereafter, every 12 h on days 1, 2, 3, 4 and 5, followed by once daily on days 6, 7, 14, 21, 28, 35, 42, and on any day of recurrent illness. During every visit, case record forms were used to document clinical assessments, history of clinical symptoms, adverse events, concomitant drug consumption, and laboratory findings.

### Data collection and laboratory assessments

#### Microscopy

During all visits, finger-prick blood smears for microscopy determination of asexual and sexual parasitaemia were collected. Two blood slides with thick and thin smears per patient were obtained at screening and enrolment. The screening slide was stained rapidly (10% Giemsa for 10–15 min) for initial reading. During enrollment (circa 1 h after screening), and all slides obtained at follow-up visits, were stained slowly with 2.5–3% Giemsa for 45–60 min. Parasites were counted against 200 white blood cells (WBC), and converted to parasite density per microliter (p/µL) assuming 8000 WBC/µL of blood. A blood smear was considered negative after examining 100 high-power fields or counting 500 WBC with no parasites seen. Each slide was read by two independent and experienced microscopists, and upon disagreement on presence of parasites or if density differed by more than 25%, the slides were subjected to a third independent and decisive reader. The mean parasitaemia of the two most concordant readings were used as final parasite densities.

#### Safety assessments

ECG machines (Sonoscape ECG IE12—Shenzhen, China) at Yombo and in Fukayosi (CardiMax FX-7402, Fukuda Denshi USA) were used to measure the QTc intervals at D0, before treatment initiation, and at D5 for all participants, corresponding to 2 h after the 12th and final dose of artemether–lumefantrine for patients allocated to the intervention arm. The ECG machines were set to provide heart rate-corrected QT interval (Bazzet’s method).

Haemoglobin concentration was measured on D0 and D7, using a portable spectrophotometer, HemoCue Hb 201+ (HemoCue AB, Ängelholm Sweden). Urine samples were collected and evaluated for haematuria at D0 and D7, using the CYBOW (Gyeongsangnam-do, Korea) urinalysis reagent test strips.

Venous blood (2 mL) were collected at enrollment and D7, to assess liver integrity by alanine aminotransferase (ALAT), aspartate aminotransferase (ASAT), serum bilirubin levels and kidney (creatinine) integrity. The samples were stored for a maximum of 48 h in the field refrigerator (4 °C) before transport to an ISO certified reference laboratory at the Bagamoyo Research and Training Unit (Ifakara Health Institute) for analysis. The biochemistry analyses were done using COBAS INTEGRA 400 plus (COBAS, USA). The values were compared with age specific normal ranges [43].

#### Pharmacokinetic analysis

For artemether–lumefantrine pharmacokinetics analysis, a total of 40 patients were randomly selected to contribute blood samples, 20 from each treatment group. Venous sample of 3 mL was collected in heparinized tubes, at two randomly selected time-points in 28 days of follow up. Venous samples were also collected from all patients with microscopy determined recurrent parasitaemia during follow-up, to assess lumefantrine plasma levels before re-treatment. Data were analysed by population pharmacokinetics modelling; the estimated parameters were used to simulate individual patient concentration time profiles from time 0 to 1008 h. The resulting profiles were used to compute maximum drug concentration in ng/mL (Cmax) and concentration at days 2, 3, 4, 5, 6, 7, 14, 21, 28, 35 and 42 for both lumefantrine and desbutyl-lumefantrine between treatment arms.

#### Lost to follow-up and patient withdrawal

Study withdrawal criteria included consent withdrawal, concomitant self-treatment with any medicine having anti-malarial properties outside the study protocol, and/or any other protocol violation. Study participants were categorized as lost to follow-up and eventually withdrawn if they missed a scheduled follow-up visit and did not attend on the successive 2 days despite efforts to trace them at their homes. A participant who missed a visit, but returned before the last day of scheduled follow-up was not considered lost to follow-up. Patients with symptoms/signs of severe disease or recurring vomiting of study medicine were withdrawn from the study and treated with parenteral artesunate 2.4 mg/kg 8 h for 24 h, followed by artemether–lumefantrine for 3 days.

### Molecular analyses

#### Dried blood spot collection

Blood samples were collected on PerkinElmer 226 filter paper (PerkinElmer, USA) during all patient visits for parasite detection and genotyping by PCR. Filter-paper blood samples were labelled, air-dried at room temperature for 3–4 h and then packed in individual Ziploc plastic bags with desiccants. The dried blood spots (DBS) were stored in room temperature until shipment to Karolinska Institutet, Stockholm, Sweden, in April 2018 where PCR analyses were conducted.

#### DNA extraction and PCR

DNA extraction from DBS collected on days 3, 5, 7, 28 and 42 was done using the chelex^®^-100 (Biorad Laboratory, USA) boiling method as previously described [44]. *P. falciparum* detection and quantification was conducted by 18 s quantitative PCR (qPCR) conducted in triplicate [45]. Samples were defined as PCR positive when any two out of the three PCR replicates were positive, i.e., having cycle quantification values below 40. PCR positive samples with parasite densities below the limit of quantification of the qPCR (1 p/µL) were assigned a parasite density of 0.5 p/µL for statistical analysis. A subset of patients underwent PCR analysis for *P. falciparum* detection for all 20 sampling time points during the 42-day study period. The subset included all patients that had recurrent parasitaemia, and 46 patients (23 from each arm) randomly selected among those that did not have recurrent parasitaemia.

#### PCR adjustment of recurrent parasitaemia

All patients with microscopy determined recurrent parasitaemia during follow-up were subjected to stepwise genotyping from paired blood sampling. Blood samples collected on the day of recurrent parasitaemia were compared with two consecutive time points at D0 and D1, by genotyping the merozoite surface proteins msp-2 and msp-1 and the glutamate-rich protein (glurp), to distinguish recrudescence (treatment failure) from reinfection (new infection) [46, 47]. Two early time points were chosen, as opposed to just one, in anticipation of natural fluctuations in density of each infecting clone, therefore, maximizing the chance of identifying all *P. falciparum* clones present in the initial infection [47]. Analysis of each marker was conducted by nested (semi-nested for *glurp*) PCR according to previously established protocols [48].

#### Genotyping for resistance markers

Genotyping of *pfmdr1* N86Y SNP was done by PCR-restriction fragment length polymorphism based methods as previously described [49, 50]. This was done in all patients with recurrent parasitaemia, on D0, D1AM and day of recurrent parasitaemia by microscopy, as well as in the D0 samples from the 46 randomly selected patients that did not have recurrent parasitaemia. Sequencing of the *pfk13* propeller region was done as previously described [51], in a subset of 96 samples that were positive in the *pfk13* nested PCR. The 858 base pairs covering the six propeller domain were sequenced, with *P. falciparum* 3D7 as a Ref. [52].

### Statistical analyses

Analyses was done using both intention-to-treat and per-protocol approaches. Proportions were calculated with 95% confidence intervals (CI) and compared by Chi-square test, whilst medians were compared by the Mann–Whitney U test. For parasite densities, geometric means were determined and compared using Wilcoxon rank-sum test. Survival analysis was done to compare post treatment prophylaxis between the treatment arms using Kaplan–Meier survival curves, and mean survival time was compared by log-rank. Kaplan–Meier analysis for PCR adjusted cure rates was also conducted. Analyses were conducted in STATA 15.0IC (StataCorp, USA); statistical significance was defined as p < 0.05.

## Results

### Patients characteristics and study flow

**Fig. 1:**
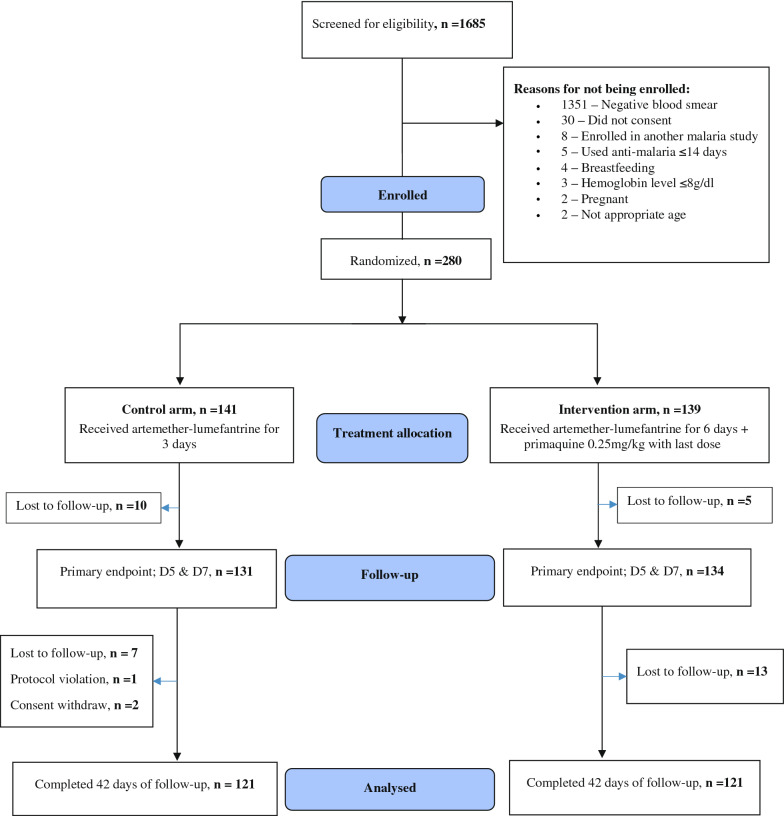
Flow of patients through the study

A total of 280 patients were enrolled in the study, 141 in the control arm and 139 in the intervention arm, of whom 121 completed 42 days follow-up in each arm (Fig. 1). Baseline demographic and clinical characteristics were similar between the arms (Table 1).

**Table 1:** Baseline demographic and clinical characteristics of patients in control vs intervention arm

Parameters	Control arm^b^ (N = 141)	Intervention arm^b^ (N = 139)
Sex, n (%) female	65 (46.0)	59 (42.5)
Age (years), median (IQR)	10 (5–17)	10 (5–14)
Age groups		
< 5 years, n (%)	48 (34.0)	35 (25.2)
5–14 years, n (%)	49 (34.8)	71 (51.1)
≥ 15 years, n (%)	44 (31.2)	33 (23.7)
Weight (kg), median (IQR)	26 (16.5–49.5)	27 (18–43)
Screening *P. falciparum* parasitaemia (p/µL), geometric mean (95% CI)	13,355 (9762–18,272)	16,986 (12,936–22,305)
Enrollment *P. falciparum* parasitaemia (p/µL), geometric mean (95% CI)^a^	15,744 (10,949–2263)	16,561 (11,812–23,219)
Patients with *P. falciparum* gametocytaemia, n (%)	3 (2.1)	1 (0.7)
Haemoglobin (g/dL), median (IQR)	12.2 (10.6–13.3)	12.0 (10.7–13.1)
Fever (axillary temperature ≥ 37.5 °C), n (%)	109 (77.3)	110 (79.1)
Axillary temperature (°C), median (IQR)	38.4 (37.6–39.0)	38.2 (37.7–39.1)

*IQR* inter-quartile range

^a^Enrollment parasitaemia was determined about 1–2 h from completion of screening just before the first dose

^b^Control arm received standard treatment with artemether–lumefantrine for 3 days and intervention arm received extended treatment with artemether–lumefantrine for 6 days with single low-dose primaquine

### Parasite clearance by microscopy and fever clearance

**Fig. 2:**
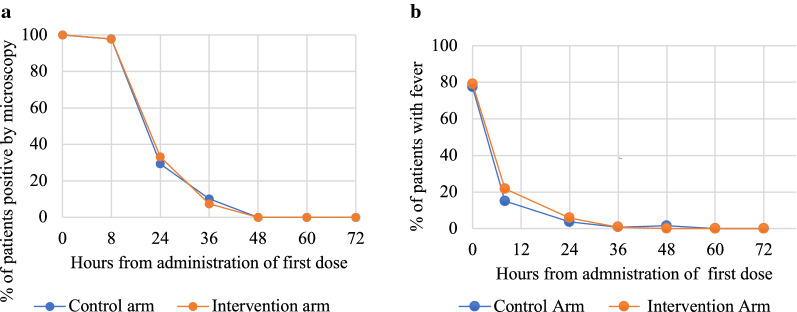
Parasite clearance by microscopy and fever clearance within treatment arms. **a** Percent of patients in each treatment arm positive by microscopy during first 72 h of treatment. **b** Percent of patients in each arm that remained febrile after initiation of artemether–lumefantrine during the first 72 h

Parasite and fever clearance were rapid and similar between the arms. By D3, all 265 patients had cleared parasites by microscopy and all patients were afebrile at 60 h (Fig. 2). There were only four patients with gametocytes at baseline. All patients were gametocyte free by D7.

### Treatment outcomes

#### Cure rates

Treatment outcomes as defined by the WHO by day 28 and 42 by treatment arm are presented in Table 2. A total of 35/242 (14.5%) patients, 21/121(17.4%) in control vs 14/121 (11.6%) in intervention arm, had microscopy determined recurrent parasitaemia, p = 0.20. After PCR adjustment of the 35 recurrent infections, 21/35 (60.0%) were determined to be reinfections [13/121 (10.7%) in the control arm and 8/121 (6.6%) in the intervention arm]; 5/35 (14.3%) were determined to be recrudescence [3/121 (2.5%) in the control arm, and 2/121 (1.7%) in the intervention arm]; and 9/35 (25.7%) were undetermined due to failure of PCR amplification [5/121 (4.1%) in the control arm and 4/121(3.3%) in the intervention arm]. PCR-adjusted cure rates at D14, D21, D28 and D42 were > 95%, with no difference between the treatment arms (Table 3). However, among patients with recurrent parasitaemia, the enrollment geometric mean parasite density was significantly higher than in patients without recurrent parasitaemia by microscopy, 14,425 p/µL (95% CI 11,077–18,786 p/µL) versus 33,764 p/µL (95% CI 17,366–65,646 p/µL), p < 0.001.

**Table 2:** Treatment outcomes by treatment arm as defined by the WHO at day 28 and 42

Endpoint (treatment outcome)	Control arm	Intervention arm
Early treatment failure, n (%)	0	0
Late clinical failure before D7, n (%)	0	0
Late clinical failure, on or after D7 n/N (%)	14/121 (11.6)	6/121 (5.0)
Due to recrudescence	1	0
Due to reinfection	11	3
Undetermined or missing PCR data n/N	2	3
Late parasitological failure, n/N (%)	7/121 (5.8)	8/121 (6.6)
Due to recrudescence	2	2
Due to reinfection	2	5
Undetermined or missing PCR data	3	1
Adequate clinical and parasitological response by D28, n/N (%)	115/121 (95.0)	118/121 (97.5)
Adequate clinical and parasitological response by D42, n/N (%)	100/103 (97.1)	107/109 (98.2)
No treatment outcome		
Lost to follow-up total, n/N (%)	17/141 (12.1)	17/139 (12.2)
Withdrew consent or protocol violation n/N (%)	3/141 (1.4)	1/139 (0.7)

**Table 3:** PCR-adjusted parasitological cure rates according to the treatment arms by Kaplan–Meier analysis

Endpoint	Control arm % (95% CI)	Intervention arm % (95% CI)
D14	100%	100%
D21	100%	100%
D28	98.4% (93.6–99.6)	100%
D42	97.4% (92.1–99.2)	98.3% (93.2–99.6)

#### Survival analysis

**Fig. 3:**
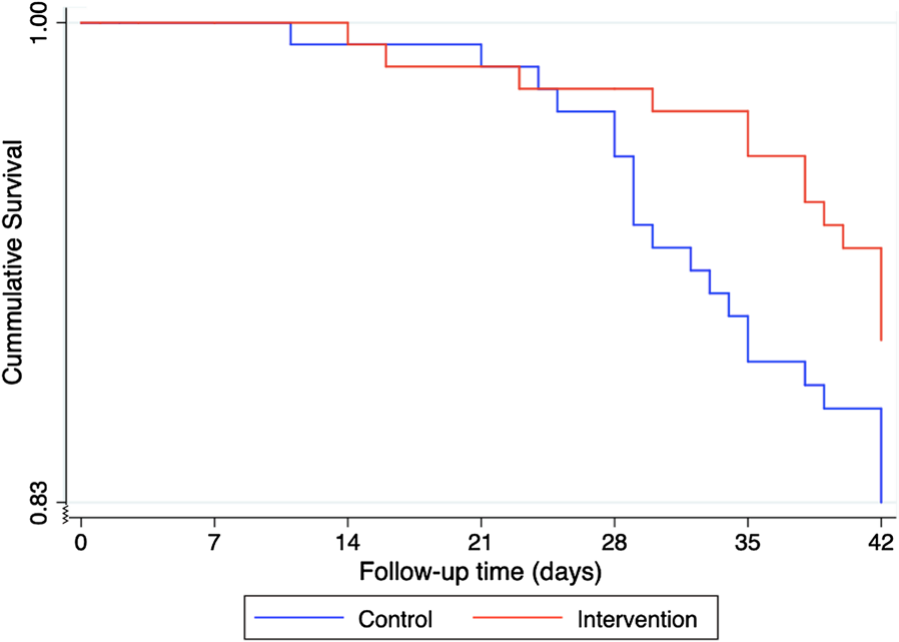
Kaplan–Meier cumulative survival curve; time to microscopy determined recurrent parasitaemia (crude cure rates)

The mean survival time to microscopy determined recurrent parasitaemia was 40.3 days (95% CI 39.4–41.2) and 41.1 days (95% CI 40.4–41.8) in the control and intervention arm, respectively (log rank p = 0.17) (Fig. 3). Survival time for 90% of the patients without recurrent parasitaemia was 34 days in the control arm, and 42 days in the intervention arm.

#### PCR determined positivity and parasite densities

**Fig. 4:**
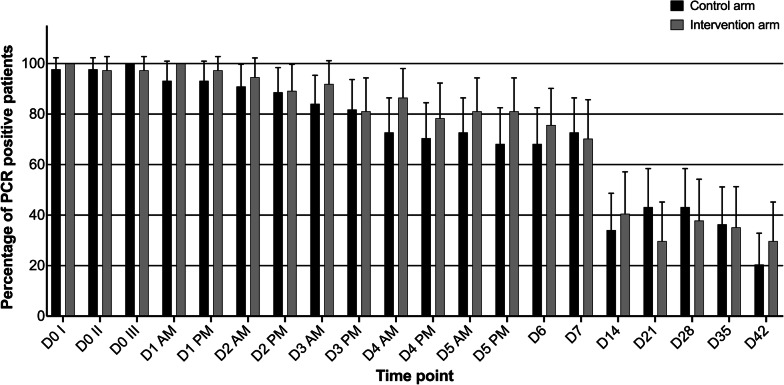
Proportion of *P. falciparum* detected by PCR among 81 patients for 20 sampling time points. Out of the 81 patients, 35 had recurrent parasitaemia by microscopy and 46 did not have recurrent parasitaemia during the 42-day follow-up period

**Fig. 5:**
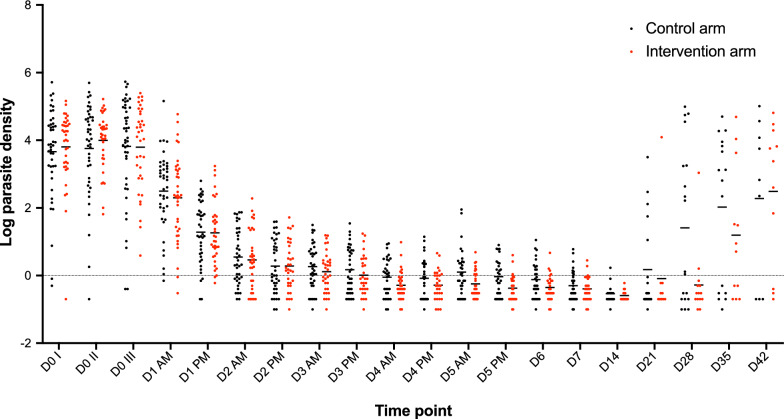
Dot plot of qPCR determined parasite densities for 81 patients for 20 sampling time points. Out of the 81 patients, 35 had recurrent parasitaemia by microscopy and 46 did not have recurrent parasitaemia during the 42-day follow-up period

There was no difference in the proportion of patients with parasites detectable by PCR and no difference in qPCR-determined parasite densities between the control and intervention arm on D5 and D7 of treatment (Table 4). Among the subset of 81 patients (30.6% of enrolled patients) that were analysed by PCR for *P. falciparum* detection at all 20 sampling time points, more than 60% remained PCR positive up to D7 in both treatment arms (Fig. 4). The majority of parasite densities reduced quickly during D0 and D1 and then remained low (< 10 p/µL) up to D42 (Fig. 5).

**Table 4:** Proportion of PCR positive patients and respective parasite densities at days 3–42 by treatment arms

Day of assessment	PCR positivity		p
	Control arm n/N (%)	Intervention arm n/N (%)	
D3	104/131 (79.4%)	113/134 (84.3%)	0.34
D5^b^	80/130^a^ (61.5%)	89/134 (66.4%)	0.44
D7^b^	71/129^a^(55.0%)	70/134 (52.2%)	0.71
D28	26/121 (21.5%)	24/122 (19.7%)	0.75
D28^c^	15/102 (14.7%)	16/111 (14.4%)	1.00
D42	20/105 (19.1%)	25/116 (21.6%)	0.74
D42^c^	15/99 (15.2%)	18/108 (16.7%)	0.85

Day of assessment	Parasite density determined by qPCR (p/µL)		p
	Geometric mean; range	Geometric mean; range	
D3	2; < 1–89	2; < 1–1051	0.98
D5^b^	1; < 1–90	1; < 1–208	0.07
D7^b^	< 1; < 1–15	< 1; < 1–115	0.29
D28	21; < 1–98,195	2; < 1–3526	0.03
D28^c^	2; < 1–3258	2; < 1–3526	0.10
D42	17; < 1–101,748	124; < 1–64,813	0.13
D42^c^	4; < 1–1395	25; < 1–60,094	0.15

^a^There was one filter paper missing on D5 and two filter papers missing on day 7 in the control arm

^b^Primary outcome variable

^c^Analysis was repeated after removing microscopy determined recurrent parasitemia patients

#### Polymorphisms in pfmdr1 N86Y and pfk13

Prevalence of *pfmdr1* N86 on D0 was 76/80 (95.0%). Among the microscopy determined recurrent infections, PCR success rate for *pfmdr1 N86Y* was 28/35 (80.0%), all of which were *pfmdr1* N86 (Table 5). No non-synonymous SNPs were detected in the *pfk13* propeller region. Five samples were observed to have a synonymous SNP in the *pfk13* propeller region, of which; four had *pfk13* C469C, and one sample *pfk13* G545G. Three of the observed SNPs were from recurrent parasitaemia samples, classified as reinfections by PCR.

**Table 5:** Genotyping *pfmdr1* N86Y and *pfk13* SNPs at D0, D1 and day of recurrent parasitaemia

	Baseline control patients^a^	Recurrent infections		
	D0	D0	D1	Day of parasite recurrence
*pfmdr1* PCR success rate n/N, %	45/46, 97.8	35/35, 100	34/35, 97.2	28/35, 80
*pfmdr1* N86 prevalence, n/N, % (95% CI)	43/45, 95.6 (84.9–99.5)^b^	34/35, 97.1 (85–100)	33/34, 97.1 (84.7–100)	28/28, 100 (87.7–100)
*pfk13* PCR success rate n/N, %	42/46, 91.3	34/35, 97.1	30/35, 85.7	26/35, 74
Prevalence of SNPs in K13 n/N, % (95% CI)	2/35, 5.9 (0.7–19.7)	2/34, 5.9 (0.7–19.7)	ND	1/26, 3.9 (0.1–19.6)

^a^Baseline control were D0 samples from 46 randomly selected patients (23 from each arm) without recurrent parasitaemia

^b^There was one patient out of 43 with mixed N/Y pfmdr1 genotype

### Pharmacokinetics results

**Fig. 6:**
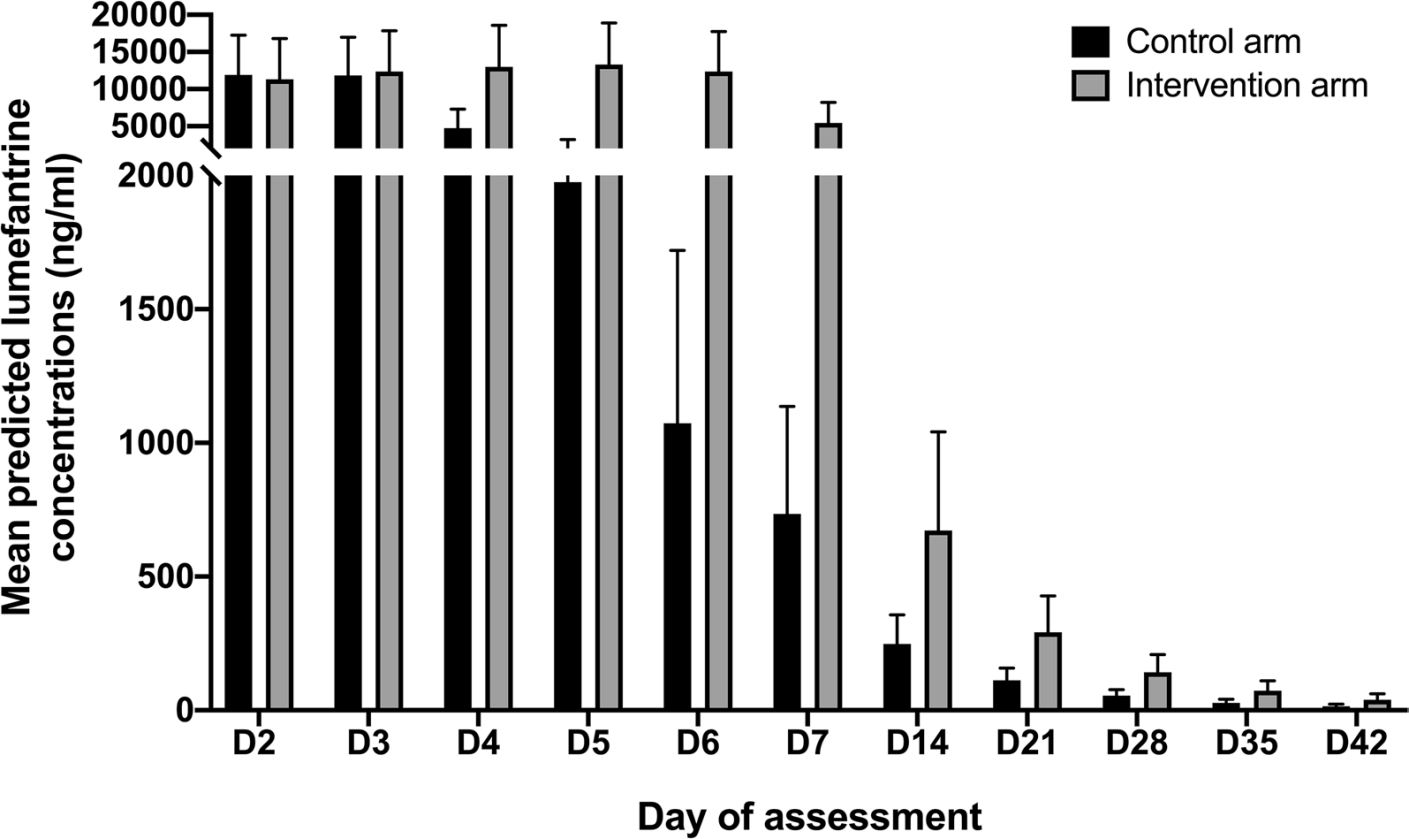
Bar plot modelling distribution of lumefantrine concentration at days 2–42 after treatment across treatment arms

Pharmacokinetics data suitable for population pharmacokinetics analysis were available from 38 of 40 patients who were randomly selected for pharmacokinetic evaluation. In addition, single plasma pharmacokinetics data were available from other 31 patients who experienced recurrent parasitaemia within the 42-day follow-up. The model predicted mean lumefantrine concentration (ng/mL) was higher in the intervention arm after D3 to D42, and the difference was highest by D7; control arm 734.8 ng/mL (95% CI 601.8–867.8) and intervention arm 5432.1 ng/mL (95% CI 4403.9–6460.3), p < 0.0001 (Fig. 6).

The mean lumefantrine Cmax in control arm was 13,747.3 ng/mL (95% CI 11,497.5–15,997.2) and in intervention arm 14,563.7 ng/mL (95% CI 11,880.5–17,246.9), p = 0.76. For desbutyl-lumefantrine, mean Cmax in control arm was 53.5 ng/mL (95% CI 44.6–62.3) and in intervention arm 73.3 ng/mL (95% CI 58.3–88.2), p = 0.05. There was no difference in mean (95% CI) Cmax for both lumefantrine and desbutyl-lumefantrine between patients with vs without microscopy determined recurrent parasitaemia within arms (Table 6).

**Table 6:** Comparing model predicted maximum serum concentration (Cmax) ng/mL for lumefantrine and desbutyl-lumefantrine by treatment arms

	Lumefantrine Cmax, ng/mL (95% CI)	p	Desbutyl-lumefantrine Cmax, ng/mL (95% CI)	p
Control arm				
Recurrent parasitaemia (n = 18)	15,322.8 (12,780.8–17,864.8)	0.05	58.8 (48.7–68.9)	0.17
Non-recurrent parasitaemia (n = 17)	12,079.2 (8308.0–15,850.5)		47.8 (32.7–62.8)	
Intervention				
Recurrent parasitaemia (n = 13)	16,197.3 (12,505.1–19,889.5)	0.15	74.6 (59.3–89.8)	0.39
Non-recurrent parasitaemia (n = 25)	13,147.9 (9162.7–17,133.2)		72.1 (46.4–97.8)	

### Safety and tolerability

There were no deaths or severe adverse events that occurred. The reported adverse events were generally mild to moderate, without significant difference between arms (23 reported adverse events in the control arm, and 22 in the intervention arm). There were no reported adverse events related to cardiac disorders. The most commonly reported adverse events in both treatment arms, irrespective of cause, were abdominal pain, asthenia, fever, nausea, vomiting and headache (Table 7). Both treatment regimens were well tolerated.

**Table 7:** Distribution of reported adverse events by treatment arm

Adverse events	Control arm	Intervention arm
Fever, n (%)	7 (30.4)	3 (13.6)
Abdominal pain, n (%)	4 (17.4)	4 (18.2)
Headache, n (%)	3 (13.0)	4 (18.2)
Asthenia, n (%)	3 (13.0)	3 (13.6)
Dizziness, n (%)	3 (13.0)	2 (9.1)
Vomiting, n (%)	1 (2.3)	3 (13.6)
Nausea, n (%)	1 (2.3)	3 (13.6)
Rashes, n (%)	1 (2.3)	0 (0)
Diarrhoea, n (%)	0 (0)	0 (0)
Total N (%)	23 (100)	22 (100)

### Haematological changes

At D7, the median (IQR) haemoglobin values were 11.5 (10.1–12.7) g/dL for the control and 11.4 (10.2–12.7) g/dL for the intervention arm, respectively (p = 0.80). The lowest recorded haemoglobin level at D7 was 6.8 g/dL from a 6 years old patient in the control arm whose baseline haemoglobin was 9.9 g/dL and baseline parasitaemia 168,720 p/µL. This patient had recovered fully by the end of follow-up period. Additional analysis of median change in haemoglobin concentration g/dL between D0 and D7 by age groups and sex for control and intervention arms was done, and demonstrated no difference (Additional file 1: Fig. S1). There was no macroscopic haematuria at D0 or at D7. Levels of haematuria determined by urinalysis strips were 1/130 (0.8%) in control and 2/132 (1.5%) in intervention arm (p = 0.39).

### Changes in the QTc intervals

The mean QTc interval at baseline was significantly different between the control and intervention arms, i.e., 422 (SD 27.5) ms and 414 (SD 20.6) ms, respectively (p = 0.004). However, on D5, the mean QTc interval were similar in the control and intervention arm, 416 (SD 25.8) ms and 416 (SD 22.7) ms, respectively (p = 0.49). The difference in mean QTc change between the control and intervention arm, − 6.4 (SD 25.6) ms and 3.1 (SD 22.1) ms, respectively, was statistically significant, 9.5 ms (p = 0.006).

### Liver and kidney function

About 36% of the patients in each of the arms showed elevated total bilirubin levels before treatment initiation, which could be associated with disease process. By the second evaluation at D7, majority of the patients had normalized their high baseline levels after treatments. Proportion of patients with elevated liver enzymes on D7 was 6/114 (5.3%) in the control and 9/101 (8.9%) in the intervention arm (p = 0.30). The corresponding proportion of patients with increased creatinine levels on D7 was 2/118 (1.7%) and 2/110 (1.8%) in the control and intervention arm, respectively (p = 0.94). These elevated levels were within the Common Toxicity Criteria (CTC) Grade 1 and these patients had elevated enzymes levels at baselines. All the patients completed study follow-up with full recovery.

## Discussion

The results of this study did not reveal a significant difference in PCR positivity or PCR determined parasite clearance between the treatment arms at both, D5 and D7. Thus, in the present context of an ACT sensitive *P. falciparum* parasite population in Bagamoyo district, Tanzania, the extended 6-day artemether–lumefantrine treatment and a single low-dose primaquine administered on 6th day, was not superior to standard 3-day artemether–lumefantrine treatment. However, microscopy determined parasite clearance, cure rates, and safety profiles were excellent and similar between the treatment arms.

Importantly, this study used consecutive day blood sampling between D3 and D7, and, thus, provided a unique opportunity to understand PCR determined parasite clearance dynamics at timepoints normally not assessed in standard ACT trials. Moreover, this study was designed and conducted, for the first time in Tanzania, as a proactive measure and part of *P. falciparum* resistance preparation. This was done by testing a new, but simple treatment strategy with old tools, which potentially could play a future role in protecting/prolonging the therapeutic life span of artemisinin-based combinations as lifesaving drugs in Africa, by simply doubling the treatment duration of artemether–lumefantrine. Given that the extended treatment had excellent efficacy and safety profile, it could be rolled out as policy in case ACT resistant *P. falciparum* enters East-Africa from South-East Asia or evolves independently, as has recently been reported in Rwanda [13]. Thus, this study contributes important evidence for the Tanzania National Malaria Control Programme, in case Tanzania is threatened by ACT resistance and there is an urgent need to consider a change in first-line treatment strategy for uncomplicated *P. falciparum* malaria. This strategy could be used while awaiting development of new efficacious anti-malarial drugs. This study’s results are similar to a smaller study conducted in pregnant and non-pregnant women in Congo, where the efficacy of both 3 and 5 days artemether–lumefantrine treatment was high, and despite increased exposure to the drug, the safety profiles were excellent [30].

### Cure rate and post-treatment prophylaxis

The PCR adjusted cure rates by D42 were similarly high between the arms, 97.1% and 98.2% in the control and intervention arm, respectively. However, after 42 days of follow-up, some non-significant differences worth highlighting were observed. First, it appears that more patients were returning with recurrent parasitaemia in the control arm compared to intervention arm, 60% and 40% respectively. Secondly, it took 34 days in the control arm, and 42 days in the intervention arm for 90% of the patients to survive without recurrent parasitaemia, i.e., a difference of 8 days despite having similar mean time to recurrent parasitaemia. The extended treatment seemed to prolong the post-treatment prophylaxis period compared to standard artemether–lumefantrine treatment. The hypothesis to this is that the difference would probably have been even more pronounced with longer follow-up periods beyond 42 days. This may be explained by the fact that the extended treatment arm received higher lumefantrine exposure, as shown in Fig. 6, which can be protective against reinfection when it is above the minimum inhibitory concentration [53]. However, this study was not designed/powered for making such a conclusion, why new studies are warranted to evaluate this in more detail.

### PCR determined parasite clearance

There was no significant difference observed in PCR positivity and PCR determined parasite clearance between the treatment arms at both D5 and D7. The D3 PCR determined positivity in this study (81.9%) is the highest recorded in Bagamoyo district since 2006 [16]. Moreover, persistent PCR positivity was observed between D3 and D7, where > 60% of patients in both arms were still positive but with very low parasite densities (the majority < 1 p/µL). By D42, more than 15% of patients in both arms were still PCR positive, excluding those with microscopy determined recurrent parasitaemia.

The highly sensitive PCR methods used in this study may partly explain the increase in D3 PCR positivity over time, as compared with previous studies from the same site [16]. Other studies in Uganda and Kenya also report high PCR positivity after ACT [16, 54, 55]. The study in Uganda linked D3 *P. falciparum* PCR positivity with the presence of asexual ring stages and mature gametocytes, but without increased risk to treatment failure [56]. The Kenyan study demonstrated that high D3 positivity was associated with treatment failure, longer gametocyte carriage, and subsequently higher transmission potential [54].

Persistent *P. falciparum* PCR positivity requires further understanding as to whether this represents viable and metabolically active parasites that were not responsive to extended treatment. Analysing clearance times of individual parasite clones may give a better insight as to whether persistent PCR positivity is linked to resistance or not [18]. Further analysis with stage specific markers to determine the relative prevalence of gametocytes to asexual parasites, may shed further light on whether this represents mature gametocytes that are not responding to artemether–lumefantrine. Although, there was no demonstrable difference in the PCR positivity between arms at D7 despite giving the single low dose of primaquine (0.25 mg/kg) in the intervention arm [56, 57].

DNA debris in circulation after parasites are killed or damaged by the drug may be another contributing factor to persistent *P. falciparum* PCR positivity. artemether–lumefantrine works synergistically with the immunity in clearing parasites through the pitting in the spleen [58, 59]. Declining malaria endemicity in Bagamoyo district from high transmission to moderate transmission [37] could be accompanied with decline in immunity in the population [60]. There is evidence of shifting malaria disease burden to older children in Tanzania [61], which was also observed in this study, where children < 5 years only accounted for approximately 30% of the recruited patients. This supports the decline in immunity in the population, and might in part explain the slower *P. falciparum* PCR determined clearance rates.

### Molecular markers for drug resistance

The prevalence of the *pfmdr1* N86 genotype linked to lumefantrine tolerance [19], was high in the parasite population both at baseline and at day of recurrent parasitaemia. This is in line with the temporal trends of selection that were observed in previous studies in Bagamoyo district [19], where the prevalence of *pfmdr1* N86 has been reported to increase significantly over time [16]. No mutations associated with artemisinin resistance were detected in the *pfk13* gene.

Very high prevalence of *P. falciparum* with lumefantrine tolerant genotypes, implores being on high alert for clinical resistance in this parasite population. The parasite response to the drug depends on host immunity and pharmacokinetics of the drug. These, together with the lack of markers of artemisinin resistance, may explain the excellent cure rate of artemether–lumefantrine despite the presence of lumefantrine tolerant genotypes in this study [62]. The saturation of tolerant genotypes for lumefantrine, provide further evidence of inverse correlation in resistance selection between arylaminoalcohols, such as lumefantrine and mefloquine, and chloroquine. Alternative treatment strategies such as use of triple ACT have been proposed to slow down the spread of drug resistance [63]. Combinations such as artemether–lumefantrine–amodiaquine are currently under investigation.

### Pharmacokinetics

The results of the pharmacokinetics modelling showed that the extended 6-day treatment improved lumefantrine exposure in patients within the intervention arm during the 42 days of follow-up. This increased lumefantrine exposure has the potential to cure the possibly resistant parasites. These results are consistent with recent findings from Congo that showed pregnant women who received 5 days of artemether–lumefantrine had higher drug exposure compared to those who received standard 3 days treatment [30]. There were no significant differences in Cmax for lumefantrine or desbutyl-lumefantrine between the two arms, which may explain why patients in the intervention arm did not have significantly more adverse events compared to control arm despite extended drug exposure. The predicted Cmax for plasma lumefantrine were not different between patients that had recurrent parasitaemia vs those without recurrent parasitaemia. This may suggest that the maximum drug concentration reached might not influence the overall treatment outcome.

### Safety and tolerability

Safety and tolerability of the extended 6-day treatment of artemether–lumefantrine and a single low-dose of primaquine was excellent. The adverse events reported were not perceived to be related with drug toxicity. Extended treatment with lumefantrine did not cause any clinically significant electrocardiographic changes, even though mean QTc change was higher in the intervention arm. These results are similar to a study in Congo of artemether–lumefantrine treatment during 5 days, where they found a weak correlation of increase in QTc and lumefantrine concentration [30]. Similar to other studies that evaluated extended artemether–lumefantrine from Myanmar and Congo, in this study, there were no biochemical or haematological changes detected that were different between the treatment arms.

## Limitations

Firstly, since artemether–lumefantrine still has excellent efficacy in the study area, a significant difference in parasite clearance by microscopy or PCR adjusted cure rate was not anticipated. Instead, the use of PCR based parasite clearance as the primary outcome was chosen. The study was powered to detect an assumed clinically meaningful difference of 15% in PCR determined *P. falciparum* positivity rate on D5. There was no data available from the study site on PCR positivity rates beyond D3, so the assumed PCR positivity rates on D5 were arbitrarily set at 20% and 5% in the control and intervention arms, respectively. However, the determined D5 PCR positivity rate (> 60% from both arms) was unexpectedly higher than assumed, indicating that PCR-determined parasite clearance requires further understanding as discussed above. Secondly, the inability to extract RNA from the DBS for distinguishing gametocytes and asexual parasites using stage specific markers, limited the understanding of the persistent PCR positivity. Thirdly, haemoglobin assessment beyond D7 was not done, and this limit thorough assessment of impact of single low-dose primaquine on extended artemether–lumefantrine regimen. Finally, since the post treatment prophylaxis in the form of time to recurrent parasitaemia was also evaluated, and this parameter could be affected by increased use of insecticide-treated mosquito nets, coverage of ITNs warrants a cautious interpretation of the results; information on individual ITN ownership and use was not collected from the study participants, however, data on ITN use were available for the region [39].

## Conclusions

Extended 6-day artemether–lumefantrine treatment together with a single low-dose of primaquine was not superior to standard 3-day artemether–lumefantrine for the treatment of uncomplicated malaria in the ACT sensitive *P. falciparum* population in Tanzania, but importantly equally efficacious and safe. Thus, this study, as part of ACT resistance preparedness in Tanzania, provides evidence that extended artemether–lumefantrine treatment could be considered as a future treatment regimen, if/when ACT resistant *P. falciparum* appears, in order to prolong the therapeutic lifespan of ACT in Africa. Moreover, the results of the study underscore the importance of studying the cause of prolonged PCR positivity after artemether–lumefantrine treatment, and its potential role in *P. falciparum* tolerance/resistance development, as well as the influence of extended artemether–lumefantrine therapy on post-treatment prophylaxis beyond day 42.

## Supplementary information

**Additional file 1: Fig. S1.** Median change in haemoglobin concentration g/dL between D0 and D7 by age groups and sex for control and intervention arms.

## Data Availability

The datasets analysed during the current study are available from the corresponding author on reasonable request.

## Abbreviations

ACT: Artemisinin-based combination therapy
ALAT: Alanine aminotransferase
AM: Ante Meridiem
ASAT: Aspartate aminotransferase
CI: confidence intervals
D: Day
DBS: Dried blood spots
ECG: Electrocardiogram
glurp: Glutamate-rich protein
HRP2: Histidine rich protein 2
IQR: Inter-quartile range
ISO: International Organization for Standardization
ITN: Insecticide treated nets
RDT: Malaria rapid diagnostic test
MS Excel: Microsoft Excel
msp-2: Merozoite surface protein
NIMR: National Institute for Medical Research
NJ, USA: New Jersey, United States of America
PAN: Other plasmodium species
PCR: polymerase chain reaction
*pfcrt*: *P. falciparum* chloroquine resistance transporter gene
*pfk13*: *P. falciparum* Kelch 13 propeller gene
*pfmdr1*: *P. falciparum* multidrug resistance gene 1
pLDH: Plasmodium lactate dehydrogenase
PM: Post Meridiem
qPCR: Quantitative polymerase chain reaction
SNP: Single nucleotide polymorphism
WBC: White blood cells
WHO: World Health Organization
