# Supplementary figures and images for: Parasite clearance, cure rate, post-treatment prophylaxis and safety of standard 3-day versus an extended 6-day treatment of artemether–lumefantrine and a single low-dose primaquine for uncomplicated Plasmodium falciparum malaria in Bagamoyo district, Tanzania: a randomized controlled trial
Source: Malar J. 2020 Jun 23;19:216. doi: 10.1186/s12936-020-03287-5 (PMC7310382; doi:10.1186/s12936-020-03287-5)

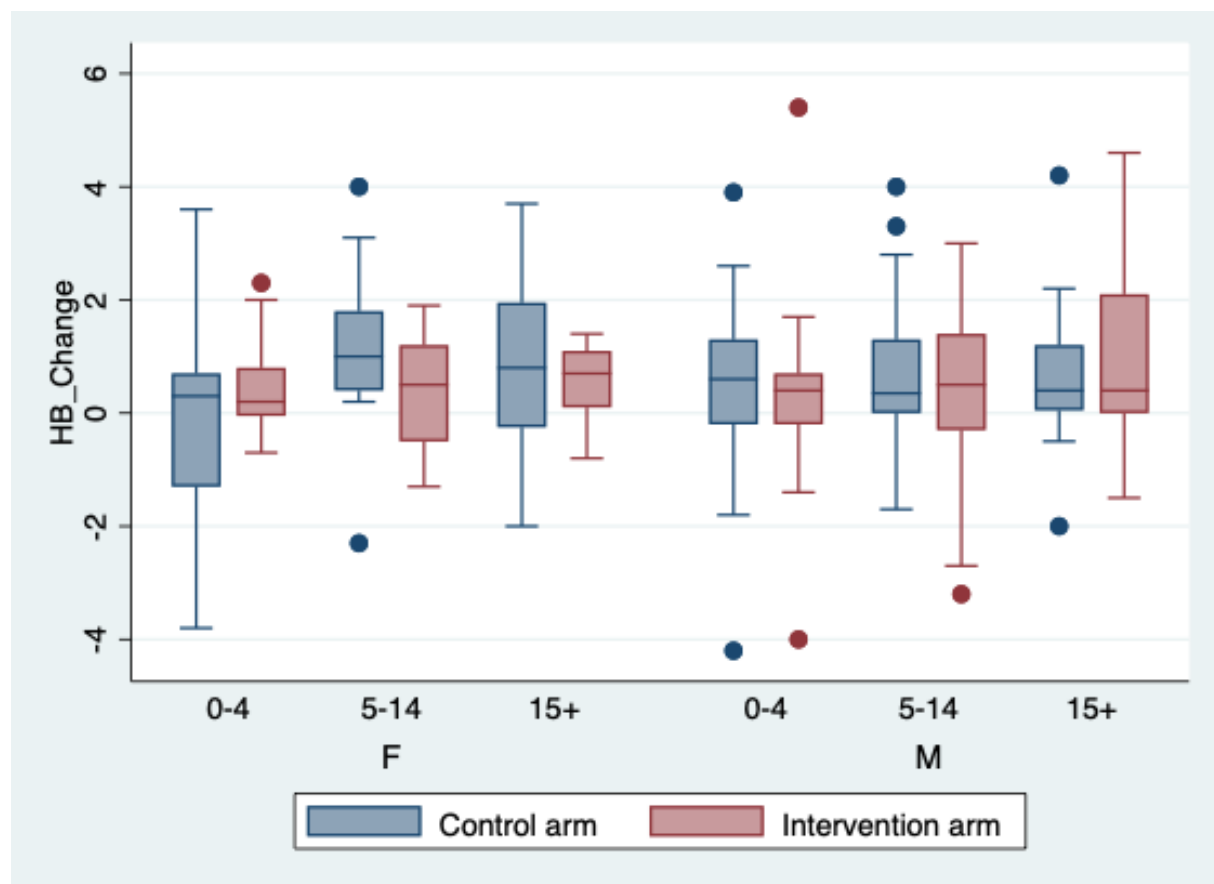

Supplement: Supplementary file 1 — Additional file 1: Fig. S1. Median change in haemoglobin concentration g/dL between D0 and D7 by age groups and sex for control and intervention arms. [file 12936_2020_3287_MOESM1_ESM.pdf]
